# Palbociclib in advanced stage hormone receptor-positive breast cancer: real-world data from a Chilean multicentre registry

**DOI:** 10.3332/ecancer.2023.1636

**Published:** 2023-11-21

**Authors:** Benjamín Walbaum, José Miguel Reyes, Pablo Rodriguez, Sabrina Muñiz, Lidia Medina, Carolina Ibañez, Tomas Merino, Mauricio P Pinto, Maria Loreto Bravo, Francisco Acevedo, José Bennett, Cesar Sanchez

**Affiliations:** 1 Department of Hematology-Oncology, Faculty of Medicine, Pontificia Universidad Católica de Chile, Santiago 8330077, Chile; 2 Dr. Sótero del Río Hospital and Healthcare Complex, Santiago 8207257, Chile; 3 Cancer Center, Clínica Las Condes, Santiago 7591047, Chile; 4 Breast Center MEDS Clinic, Santiago 7550557, Chile; 5 Oncology Department, Clínica IRAM, GESMED, Santiago 7630370, Chile; 6 ‘Nuestra Señora de la Esperanza’ Cancer Center, UC CHRISTUS Healthcare Network, Pontificia Universidad Católica de Chile, Santiago 8330032, Chile; 7 Support Team for Oncological Research and Medicine (STORM), Santiago 7510123, Chile

**Keywords:** advanced breast cancer, hormone receptor-positive, CDK4/6 inhibitors, endocrine therapy, survival

## Abstract

**Background:**

The addition of cyclin-dependent kinases inhibitors (CDKi) to endocrine therapy (ET) as the first- or second line treatment improves progression-free and overall survival (OS) in hormone receptor-positive, HER2 negative (HR+/HER2-) advanced stage breast cancer (ABC). Our study compared survival rates and prognostic factors in Chilean patients that used palbociclib as first or subsequent (≥second) lines of treatment in a real-world setting.

**Methods:**

Our retrospective population-cohort study included HR+/HER2- ABC patients. We calculated 5-year OS and performed a multivariate analysis to determine prognostic factors.

**Results:**

A total of 106 patients were included. Median age was 49 years (19–86), 28.3% (30) had de novo stage IV disease; 63% received palbociclib with ET as first line, 54% of them with aromatase inhibitor over fulvestrant. Median OS for the entire cohort was 99 months and 5-year OS was 69%. Patients that received first line palbociclib had a 5-year OS of 89% versus 43% for ET monotherapy or ≥second line palbociclib (*p* = 0.0062). Multivariate analysis showed that the year at diagnosis and CDKi timing (first line versus ≥second line) were significantly associated with OS.

**Conclusion:**

Our real-world data show that first-line CDKi + ET provides a statistically significant benefit in OS versus ≥second line in HR+/HER2- ABC patients.

## Introduction

Worldwide, breast cancer (BC) is the most common female cancer. In Chile, BC became the leading cause of cancer death in 2020 claiming over 1,600 lives every year [1]. As in other Latin American countries, Chile has yet to implement a formal BC screening program, which might partly explain the high rates of locally advanced BC observed at diagnosis. In fact, these can reach up to 20% for stage III hormone receptor-positive (HR+) BC [2]. Previous studies demonstrate that >45% of these patients will recur after a 20-year follow-up period [3] and with approximately 6% of patients being diagnosed with *de novo* stage IV disease [4], the result is a growing burden of HR+/HER2-advanced-stage BC (ABC) patients, representing 54% of all stage IV cases in Chile [5].

Cyclin-dependent kinases 4 and 6 (CDK4/6) play a key role in cell-cycle progression and consequently have become relevant targets for BC treatments, especially in HR+/HER2- cases. This has led to the development of several specific CDK4/6 cyclin-dependent kinase inhibitors (CDKi) against these kinases that improve BC patients’ survival [6]. Despite their demonstrated efficacy, Latin American studies reporting the usage of CDKi in real-world settings are very scarce. In this context, the Ibrance Real World Insights (IRIS) study and the RENATA trial [7] are perhaps some of the few examples with real-world data (RWD) from Colombia and Argentina [8]. To date, no local reports have been published in Chile.

Palbociclib was the first CDKi to be approved in Chile and started its use in 2016. Shortly afterwards, ribociclib and abemaciclib were also approved and became part of the standard treatment for HR+/HER2- ABC patients. Despite this, there is still not enough data to compare or confirm the superiority of adding CDKi to first-line endocrine therapy (ET) versus subsequent lines of treatment. The prospective phase III SONIA-trial recently reported no significant benefit of adding CDKi to first-line treatment [9]. Given the prior lack of evidence, and its high costs, the addition of CDKi to first-line hormonal therapy in our country is restricted to patients with private health insurance, whereas patients in the Chilean public health system can only receive palbociclib after progression to first-line ET [10]. Herein, we compared the impact of palbociclib in overall survival (OS) either added to first-line ET or to subsequent lines of treatment in a real-world setting.

## Methods

### Study design and ethics approval

This retrospective population-cohort study was conducted in collaboration with the Cancer Center of Pontificia Universidad Católica de Chile, which is an Academic Private Center (AC), GESMED Cancer Center, and Clínica las Condes Cancer Center (CLCC). All private institutions are in Santiago, Chile. Our procedures respected ethical standards by the Declaration of Helsinki (updated in 2013) and were reviewed and approved by a Human Research Ethics Committee at our institution (School of Medicine, Pontificia Universidad Católica de Chile, Santiago, Chile; approval resolution number: 200303006).

### Patients and clinical data

Data on HR+/HER2- ABC patients treated with palbociclib plus ET were retrospectively collected from three centres (AC, GCC and CLCC), registering clinical and pathological variables such as age, body mass index (BMI) at diagnosis, stage at initial diagnosis (TNM AJCC 8th version), HR expression and Ki67 status, initial metastasis site (visceral or non-visceral) and ET used accompanying palbociclib (fulvestrant or aromatase inhibitors (AI)); OS was defined as the time between the diagnosis of advanced disease (metastatic or locoregionally recurrent disease not amenable to curative treatment) and death (by any cause). Luminal A subtype was defined as high oestrogen receptor (ER) and the progesterone receptor (PR) expression ≥20%, with a histological grade (HG): 1 or 2 and Ki67 <20%. The Luminal B subtype was defined as ER+ and/or PR+ <20%, HG: 3, and Ki67 ≥20% [11].

### Statistics

Continuous variables are presented as medians and ranges, while categorical variables are presented as frequencies and percentages. The chi-square test was used to analyse the relationship between categorical variables. Survival analysis was carried out using the Kaplan­­­-‑Meier method and log-rank test or Wilcoxon to compare survival distributions. Survival outcomes were compared between patients receiving CDKi treatment in the first line versus subsequent lines. We performed a univariate and multivariate logistic regression analyses to identify clinical and pathological risk factors associated with OS. All *p*-values were two-sided and *p* < 0.05 was considered statistically significant. All statistical analyses were performed using SAS statistical software version 16.1 (SAS Institute Inc., Cary, NC, USA).

## Results

**Figure 1.**
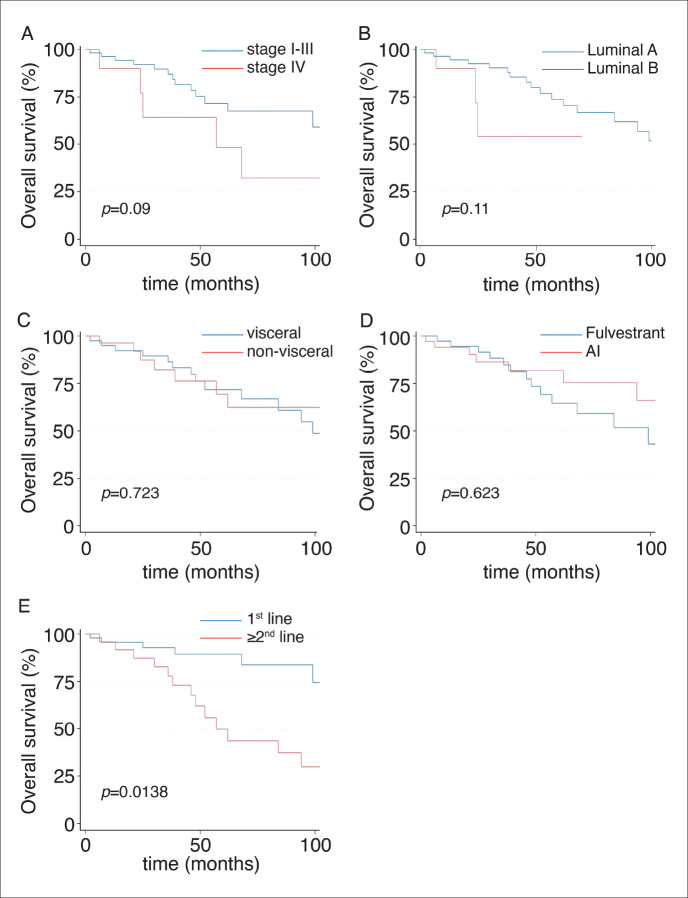
OS of patients. (A): By stage. (B): By luminal subtype. (C): By presence or absence of visceral disease. (D): By type of ET. (E): By line of treatment.

The main clinical characteristics of patients in our study (*n* = 106) are summarised in Table 1. Briefly, the median age was 49 years. (range: 19–86); 49% of patients were from an AC. As expected, most patients (79%) were luminal A, and only 30% had *de novo* stage IV disease. Also, 64% received palbociclib plus ET as the first line (Table 1); 55% with AI and 45% with fulvestrant. Similarly, AI was more frequently used as first-line treatment accompanying CDKi compared to second- or subsequent lines of treatment (67.2% versus 31.5%, respectively; *p* = 0.001, Table 2). Next, we sought to determine which variables were associated with OS in our cohort. Figure 1 shows that stage (I–III versus IV; Figure 1A), BC subtype (Luminal A versus B; Figure 1B), presence/absence of visceral disease and type of ET (fulvestrant versus AI) were not associated with significant changes in OS. In contrast, first-line palbociclib-combined treatments had significantly better OS compared to patients receiving treatment in ≥second lines (Figure 1E; *p* = 0.0138). The impact of first-line versus ≥second line was further confirmed by significant differences in 5 years. OS and median OS (89% versus 43% and 111 months versus 52 months; respectively, *p* = 0.0062, Table 3).

Lastly, we performed a multivariate analysis that included age, initial stage, visceral disease, line of palbociclib use (first versus ≥second line) and year of diagnosis. Among these, palbociclib indication and year of diagnosis were the only ones with statistical significance (Table 4).

## Discussion

To our knowledge, this is the first multicentre, registry-based Chilean report of RWD on the use of combined ET plus CDKi. Our findings indicate that over 60% of HR+/HER2- ABC patients in our cohort received ET combined treatments as first line, in most cases combining AI plus palbociclib. Also, 30% of patients received CDKi after progression, with a significant difference in 5 years. OS favouring first-line treatment over second- or subsequent lines of treatment (89% versus 43%; *p* = 0.0062). The survival benefit of first-line treatment was further confirmed by our multivariate analysis (Table 4).

As pointed out earlier, the SONIA-trial did not find differences in OS between patients that received CDKi as first- or second line of treatment. This was regardless of a significant initial benefit in progression-free survival for the first line. However, when we compared the clinical characteristics of both these cohorts, we noted that patients in our cohort were younger, with a median age of 49 versus 64 years in the SONIA trial, with 87% of women defined as postmenopausal [9], whereas almost a half of patients in our cohort were either pre- or perimenopausal. In this regard, the right choice trial [12] recently demonstrated that this subset of patients displays a significant and clinically meaningful benefit derived from first line combined therapy. Secondly, our cohort included patients that could have progressed while on adjuvant treatment, with two-thirds having stage IV after recurrence, and the majority being diagnosed in year 2015 (mode) and recurring in 2019 (mode); thus, considering the standard use of at least 5 years of adjuvant hormonal therapy, probably still on ET. Plus 45% of patients received fulvestrant as first line, and given guideline recommendations, probably concentrating patients that recurred before 1 year of completing adjuvant ET. Consequently, our cohort included a high proportion of endocrine resistant BC, whereas the SONIA trial [9] excluded patients that progressed before 12 months of completing adjuvant ET.

Previously, our research group reported that Luminal A patients had significantly better median OS versus Luminal B (63 months versus 33 months; *p* = 0.004) in a similar cohort of ABC patients (39% of *de novo* stage IV and 61% of systemic recurrences) [13]. Notably, none of these patients received CDKi. Here, we confirm this difference between Luminal A and B tumours, however in this case, differences did not reach statistical significance. This lack of significance could be at least partially explained by the introduction of CDKis. Other factors might include some of the specific clinical characteristics of this cohort such as age at diagnosis, as mentioned above, median age at diagnosis was 49 years, also >70% were Luminal A. In addition, patients that initially received chemotherapy as part of their treatment were excluded from the study; this subset of patients usually include those with a high burden of disease or with visceral crisis. Furthermore, all patients were treated at private health institutions and consequently had better access to high-cost drugs and support of care units. Also, patients that receive medical care at private centres usually display a lower burden of disease at diagnosis. Finally, our staging strategies have improved over time, with better imaging, which may account for a stage migration, a phenomenon also known as Will Rogers Phenomenon [14] that could also have played a role. Collectively, all these factors may have contributed to higher survival rates in our cohort versus those reported by pivotal palbociclib trials such as PALOMA-2 or PALOMA-3 [15, 16]. Nevertheless, our cohort included a high proportion of patients with visceral disease and up to 38% of them received CDKi either as second- or subsequent line of treatment. Therefore, this subset included patients that displayed some degree of endocrine resistance. In terms of treatment strategies, we did not observe any differences between first-line AI and first-line fulvestrant -within the endocrine sensitive setting- which is in line with the results reported by the PARSIFAL trial [17]. Similarly, the presence or absence of visceral metastasis did not show differences in terms of survival, confirming the safety and efficacy of CDK4/6i in visceral disease, and downplaying the relevance of early chemotherapy in this subset of patients [18].

Although the definition of BC subtypes based on Immunohistochemistry (IHC) has several limitations, it is evident that patients with a lower expression of HRs, higher Ki67 and higher HG have poorer prognosis across all BC stages [19]. A study by Prat *et al* [20] performed a PAM50-based analysis of tumour samples obtained from the phase III MONALEESA trials (2, 3 and 7), and compared the efficacy of ribociclib among intrinsic BC subtypes. Investigators reported that 46.8% of tumours were Luminal A while 24% were Luminal B. In line with our findings, this study found that Luminal B tumours had a 44% higher risk of progression versus Luminal A tumours (RR: 1.44). Also, all subtypes exhibited benefits derived from the use of CDKi, except for the basal-like subtype.

Following the approval of CDKi for ABC, a couple of Latin American studies such as the IRIS [8] and the RENATA [7] have reported RWD regarding the efficacy of these treatments. Comparatively, we observe similarities but also some differences with our results. First, a similar proportion of patients in these studies received first-line combination therapy, 65%, 59% and 64% for the IRIS, the RENATA and the current study, respectively. Within this subset, 35% and 45% of participants used fulvestrant as accompanying ET in the RENATA trial and in our cohort, respectively. Among differences, perhaps the most evident is the age. While the RENATA and the IRIS trials report 57 and 64 years, respectively, our cohort was considerably younger with a median age of 49 years. It is also noteworthy that in line with our results, the P-REALITY-X [21] confirmed the impact of early administration of CDKi. However, only a half (50%) of patients that received first-line ET in this study also received CDKi in subsequent lines of treatment, underscoring the importance of local RWD which may account for the differences observed in population’ characteristics.

Our study has several limitations, mainly derived from its retrospective nature. Firstly, it has a relatively low number of patients. Also, patient information on prior ET in the adjuvant setting, endocrine resistance, subsequent treatments, including chemotherapy use, toxicities and long-term follow-up were not available and might be a potential bias. Second, considering the median age of our cohort (49 years), it is reasonable to speculate that at least a considerable proportion of them could be premenopausal; however, since data were collected retrospectively, no other reliable information on menopausal status was available. Despite this, our work delivers a first overview of the usage of ET in Chile, confirming the clinical impact of CDKis and leaving an open question regarding the potential survival benefit of first-line combined therapies in younger patients. Our study also reinforces the importance of RWD particularly within Latin America, where access disparities are extremely relevant and limit our interpretation of data coming from abroad [22] particularly when comparing sequence strategies if we consider patient attrition, with many women losing the window of opportunity for CDKi use. Finally, it provides local evidence to help define treatment guidelines, specifically in this case on whether CDKi should be available as first line treatment, given that today its reimbursement is restricted nationwide to patients progressing after a first line endocrine monotherapy.

## Conclusion

First-line palbociclib (CDKi) in combination with either AI or fulvestrant is associated with a significant benefit in OS (versus ≥second line) in a relatively young cohort of HR+/HER2- ABC patients in our real-world setting.

## Conflicts of interest

The authors declare no conflicts of interest.

## Funding

The authors declare that no funds, grants, or other support were received during the preparation of this manuscript.

## Figures and Tables

**Table 1.** Clinical characteristics of patients.

Variable	
Median age (year (range))	49 (19–86)
Median BMI (kg/m^2^(range))	29 (20.3–54.2)
	***n* (%)**
Stage at diagnosis	
I	15 (15)
II	33 (33)
III	22 (22)
IV	30 (30)
BC subtype	
Lum A	78 (79)
Lum B	21 (21)
Site of metastasis	
Visceral	59 (60)
Non-visceral	40 (40)
CDKi line of treatment	
First	67 (64)
≥Second	38 (36)
Type of ET	
AI	58 (55)
Fulvestrant	48 (45)
Enrolling centre	
AC	48 (49)
GCC	26 (27)
CLCC	23 (24)

yr.: years; BMI: body mass index; BC: breast cancer; Lum: luminal; CDKi: cyclin-dependent kinase inhibitor; ET: endocrine therapy; AI: aromatase inhibitor; AC: academic centre; GCC: GESMED cancer centre; CLCC: Clinica Las Condes cancer centre

**Table 2.** Type of accompanying ET by line of treatment.

Line of treatment	Fulvestrant	AI	*p*-value
First	32.8	67.2	0.0001
≥Second	68.4	31.6	

AI: aromatase inhibitor

**Table 3.** Five year-OS in patient subsets.

Subset of patients	5 years-OS (in %)	*p*-value
Stage IV patients	66	-
Recurrent disease	72	-
Stage IV + recurrent	69	-
By subtype (ABC)		
Lum A	70.5	0.15
Lum B	54	
By line of treatment		
First-line	89	**0.0062**
Second line	43	
By site of metastasis		
Visceral	71	0.45
Non-visceral	62	
By type of ET		
Fulvestrant	64	0.19
AI	75	
All patients	86	-


yr.: years; ABC: advanced stage breast cancer; Lum: luminal; ET: endocrine therapy; AI: aromatase inhibitor

*p*-values in bold <0.05

**Table 4.** Univariate and multivariate analysis of OS.

	Univariate		Multivariate	
Variable	HR (CI: 95%)	*p*-value	HR (CI: 95%)	*p*-value
Age	0.97 (0.93–1.01)	0.28	-	-
Initial stage				
I–III	Ref.	0.212	-	-
IV	1.83 (0.70–4.73)			
Subtype	2.46 (0.68–8.96)	0.164	-	-
Ki67	1.01 (0.96–1.03)	0.945	-	-
Line of treatment	3.01 (1.31–6.89)	**0.0001**	2.36 (1.01–5.51]	**0.047**
Visceral disease	1.35 (0.60–3.06)	0.46	-	-
Year of diagnosis	1.33 (1.17–1.53)	**0.0001**	1.31 (1.14–1.50)	**0.0001**

HR: hazard ratio; CI; confidence interval; Ref.: reference

*p*-values in bold <0.05
